# Short-Interval Two-Stage Treatment of a Comminuted Open Tibial Diaphyseal Fracture

**DOI:** 10.7759/cureus.103016

**Published:** 2026-02-05

**Authors:** Haitham Alahmar

**Affiliations:** 1 Orthopaedics and Trauma, Idlib University, Idlib, SYR

**Keywords:** comminuted, diaphyseal, open tibial fracture, short-interval, two-stage treatment

## Abstract

Open comminuted tibial fractures caused by gunshot injuries remain challenging due to the high risk of infection, bone loss, and prolonged functional impairment. We report the case of a young male patient who sustained a severe open comminuted tibial diaphyseal fracture with a large segmental bone defect following a gunshot injury. The patient was managed using a short-interval two-stage approach, consisting of early distraction osteogenesis with an external fixator followed by timely conversion to intramedullary fixation combined with autologous bone grafting. This strategy allowed effective reconstruction of the bone defect, achievement of solid union, and early restoration of limb function without major complications. This case highlights the potential benefits of minimizing the interval between treatment stages in the management of complex open tibial fractures.

## Introduction

Open tibial diaphyseal fractures resulting from high-energy mechanisms-particularly gunshot injuries-represent one of the most complex challenges in orthopedic trauma surgery. These injuries are frequently associated with severe soft-tissue damage, contamination, extensive comminution, and segmental bone loss. As a result, reported rates of infection, delayed union, and non-union remain high, reaching up to 20%-30% in severe open fractures, especially in war-related settings [1,2]. The subcutaneous location of the tibia and its limited soft-tissue envelope further increase vulnerability to infection and reconstruction failure.

Management of large post-traumatic tibial bone defects remains controversial. Traditional reconstructive options include prolonged external fixation, staged bone grafting, vascularized fibular grafts, and limb reconstruction techniques. Among these, distraction osteogenesis, a biological process in which new bone is generated by gradual mechanical distraction, has become a cornerstone technique for managing segmental defects. Using circular external fixation systems such as the Ilizarov frame, bone transport allows gradual movement of a bone segment across a defect until it reaches the distal fragment, where union occurs at the docking site [3,4]. This method enables simultaneous correction of bone loss, limb-length discrepancy, and deformity while supporting infection control.

Despite its effectiveness, prolonged external fixation is associated with significant morbidity. Reported external fixation times commonly exceed 9-12 months, with pin-tract infection rates ranging from 30% to 80%, as well as joint stiffness, patient discomfort, and delayed functional recovery [4,5]. These limitations have prompted interest in combined strategies aimed at reducing the duration of frame dependence.

One such strategy involves conversion from external fixation to intramedullary nailing after completion of bone transport. Early intramedullary conversion may provide improved mechanical stability, facilitate earlier weight-bearing, and enhance patient comfort. However, this approach remains controversial due to concerns regarding infection recurrence, insufficient regenerate maturation, and docking-site non-union. While some studies have reported acceptable alpha union rates and reduced external fixation times, evidence remains limited, heterogeneous, and largely derived from small series [6,7]. Moreover, the optimal timing of conversion-particularly short-interval conversion-has not been clearly defined, and data specific to war-related injuries are scarce.

In this report, we present a case of a young patient with a severe war-related comminuted open tibial diaphyseal fracture complicated by a large segmental bone defect. The patient was treated using a short-interval two-stage approach, consisting of initial bone transport with an Ilizarov external fixator followed by early conversion to intramedullary nailing with autologous cancellous bone grafting at the docking site. This case aims to illustrate the feasibility and potential advantages of minimizing the interval between these two stages, while acknowledging the limitations inherent to a single-case observation and the need for further investigation.

## Case presentation

**Figure 1:**
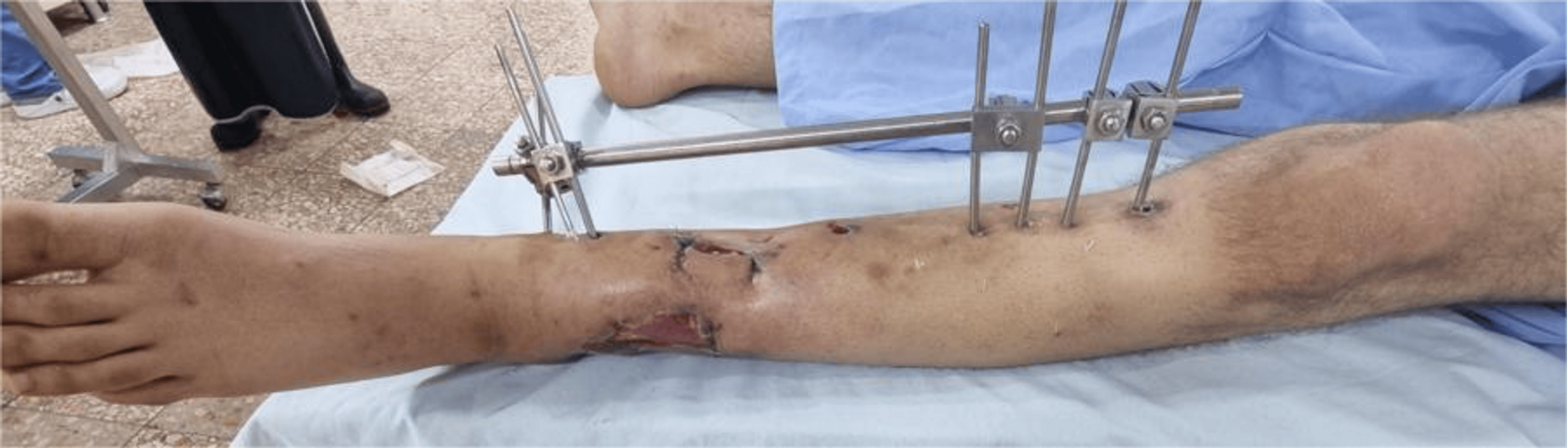
Photograph of the left leg showing the entry and exit gunshot wounds at the distal third of the leg. The entry wound is anteromedial, measuring approximately 3 cm, while the exit wound is lateral and irregular, measuring approximately 2-6 cm, consistent with a high-energy gunshot injury.

A 22-year-old male university student sustained a gunshot injury to the distal third of the left leg, slightly below the knee. The entry wound (~3 cm) was anteromedial at the distal third of the leg. The exit wound was ragged (approximately 2-6 cm), lateral at the same level (Figure 1).

**Figure 2:**
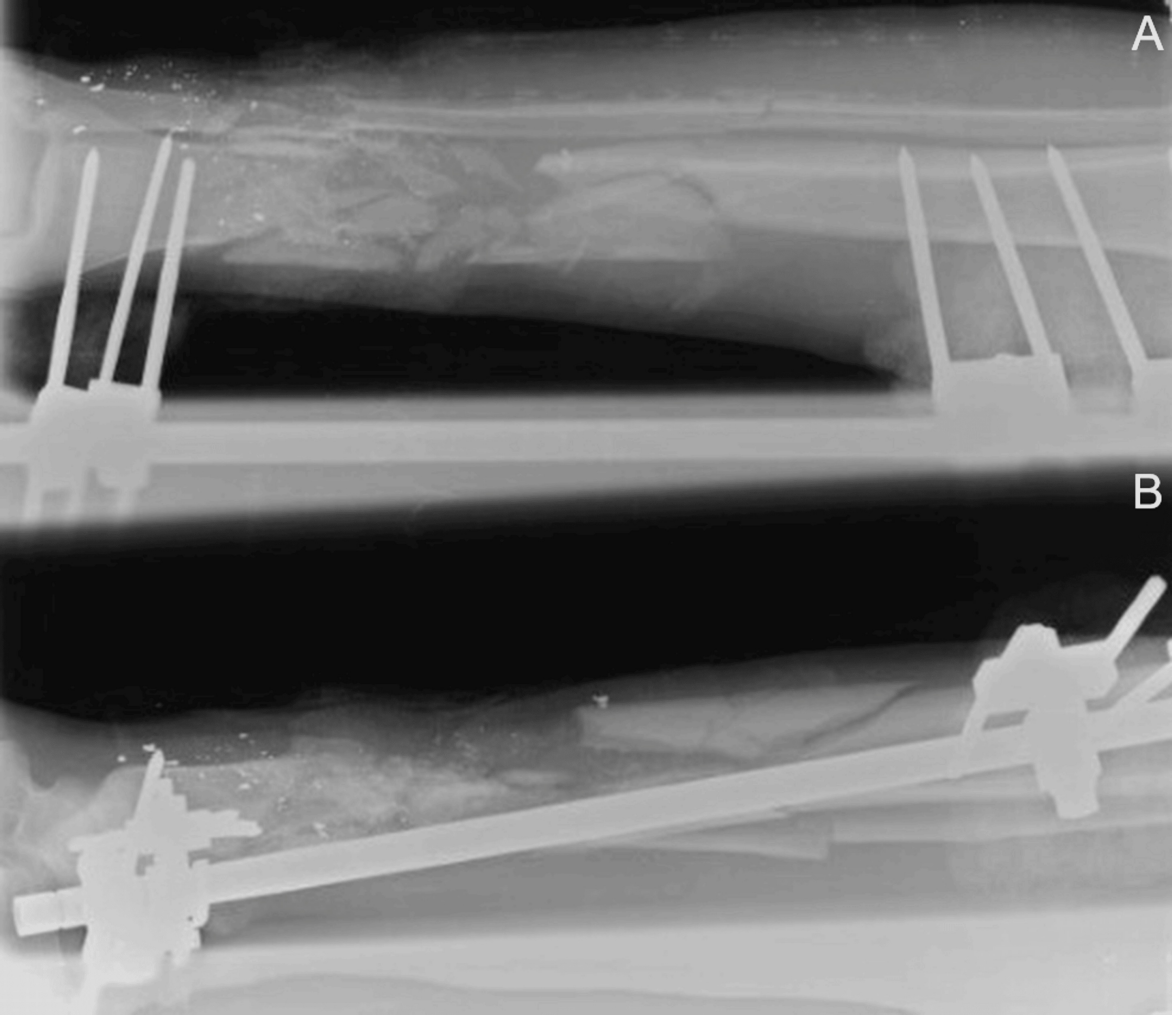
(A, B) Anteroposterior and lateral radiograph demonstrating stabilization of a severely comminuted tibial diaphyseal fracture.

Thorough clinical examination revealed intact motor and sensory function of the nerves, with no evidence of tendon injury. Arterial and venous circulation was preserved throughout the lower limb. Plain radiographs showed a comminuted diaphyseal fracture of the distal third of the tibia with an associated ipsilateral fibular fracture (Figure 2).

The patient was transferred to a nearby hospital, where he received prompt emergency care and stabilization of his general condition. He was taken to the emergency operating room, where thorough wound irrigation and limited debridement of devitalized tissues were performed. An AO external fixator was applied to stabilize the fracture (Figure 2), and the wounds were approximated. Sterile dressings were applied over the wounds and around the pin sites. The patient was admitted to the inpatient ward for neurological, vascular, and general condition monitoring.

The patient was discharged home on the day following the emergency surgical procedure. He was prescribed broad-spectrum intravenous antibiotics for three days and instructed to perform daily dressings using povidone-soaked gauze for the wounds and around the pin sites.

Follow-up of a referred case

The patient returned on the fourth day post-injury to the orthopedic outpatient clinic at Idlib University Hospital, where it was decided to admit him to the orthopedic ward. Preoperative anesthesia consultation and laboratory workup were completed, and two units of cross-matched blood were prepared for surgery.

The patient was taken to the elective orthopedic operating room, where spinal anesthesia was administered, and a thigh tourniquet was applied [1]. The external fixator was removed, and an anteromedial approach was performed. Thorough surgical debridement was carried out, including excision of all comminuted bone fragments from the tibia, followed by approximate wound closure [2].

**Figure 3:**
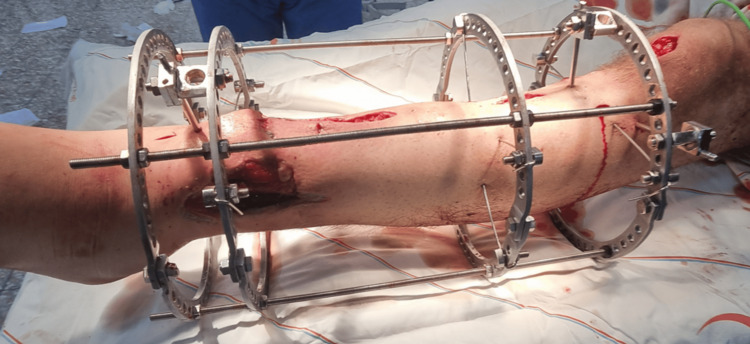
Intraoperative photograph demonstrating stabilization of a complex tibial shaft injury using an Ilizarov circular external fixator with multiple rings and tensioned wires to provide secure fixation and allow for staged limb reconstruction.

A second incision was made at the proximal anteromedial aspect of the left leg, and a proximal tibial osteotomy was performed. The three bone segments were stabilized using an Ilizarov external fixator: the proximal segment with a ring and wires, the middle segment with a ring and wires, and the short distal segment with two rings and wires (Figure 3) [3].

Proper wound closure was achieved. Intraoperatively, acute translation of the middle segment by approximately 2 cm was performed, and standardized dressings were applied [4].

The patient was admitted to the orthopedic ward for clinical monitoring and was discharged home on the day following the surgical procedure. He was instructed to perform daily wound dressings and was prescribed intravenous broad-spectrum antibiotics for five days [5], along with a strong analgesic for pain management.

**Figure 4:**
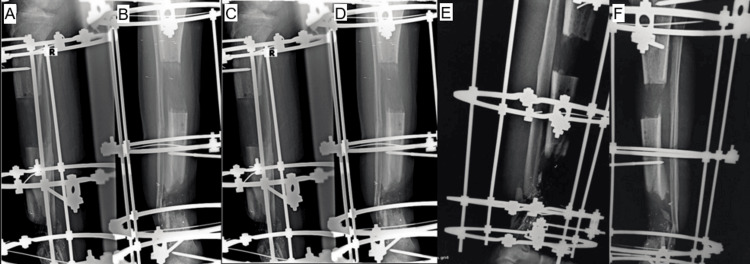
(A-F) Anteroposterior and lateral radiographs show a segmental tibial diaphyseal defect managed with an Ilizarov circular external fixator. The images demonstrate progressive distraction osteogenesis and bone transport for reconstruction of post-traumatic bone loss as part of staged limb salvage treatment.

Bone transport was initiated on postoperative day 10 at a rate of 1 mm per day for the middle segment in the distal direction (Figure 4) [4,6,7]. Oral antibiotics were prescribed for two weeks as coverage during the distraction phase.

**Figure 5:**
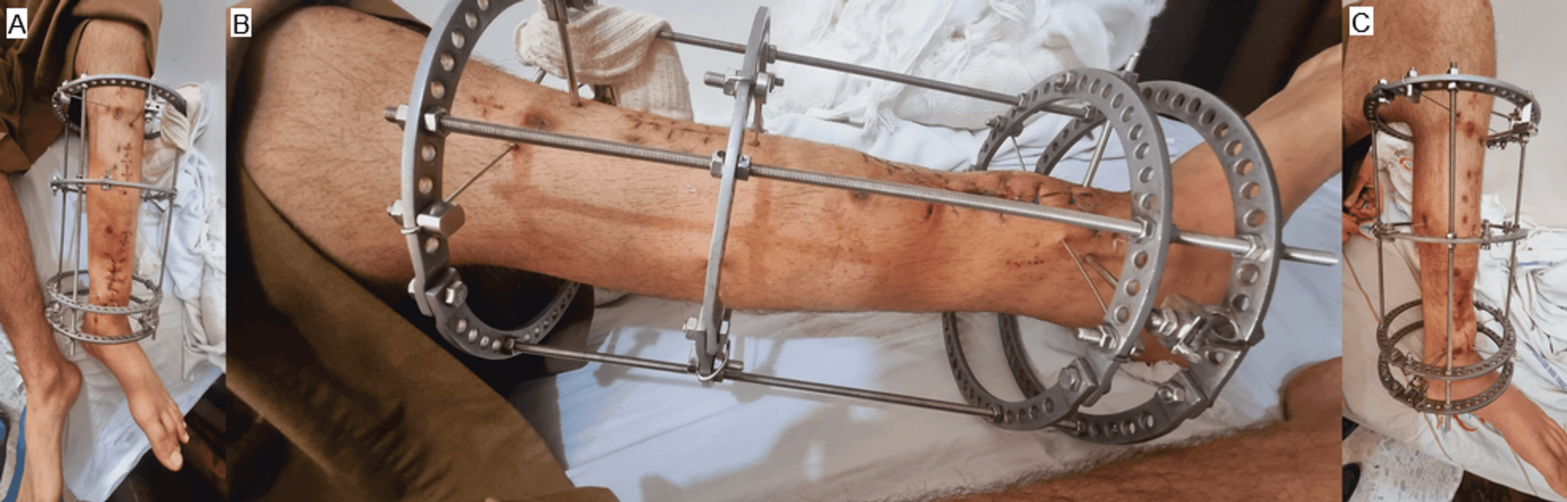
(A-C) Clinical images demonstrating postoperative stabilization of the tibia using an Ilizarov circular external fixator with multiple rings and tensioned wires. The construct provides rigid fixation and supports staged limb reconstruction following severe post-traumatic injury.

The following figure demonstrates the gradual distal sliding of the intermediate bone segment, driven by the second proximal ring moving downward to fill the segmental bone defect (Figure 5). 

The patient was prescribed oral amoxicillin-clavulanate for four days to complete a total antibiotic course of seven days. This agent was selected because of its broad-spectrum activity against Gram-positive and Gram-negative bacteria commonly associated with open fractures, its favorable oral bioavailability, and its suitability for continuation of treatment in the outpatient setting after clinical stabilization during the distraction phase.

**Figure 6:**
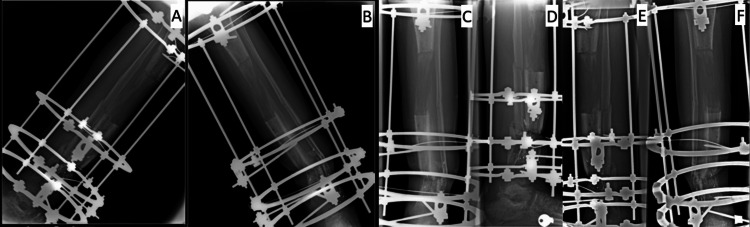
(A-F) Anteroposterior and lateral radiographs showing completion of the bone transport phase, with contact between the transported middle segment and the distal tibial fragment and early callus formation at the distraction site (refers to the junction where the transported bone segment meets the stationary bone fragment during bone transport. It represents the site of final bone contact and consolidation. This area is biomechanically and biologically critical and may require compression, bone grafting, or additional stabilization to achieve union.

On day 100 of bone distraction, the middle bone segment reached and contacted the distal segment (Figure 6), with the initiation of callus formation observed at the distraction site [8].

Second stage of surgery

**Figure 7:**
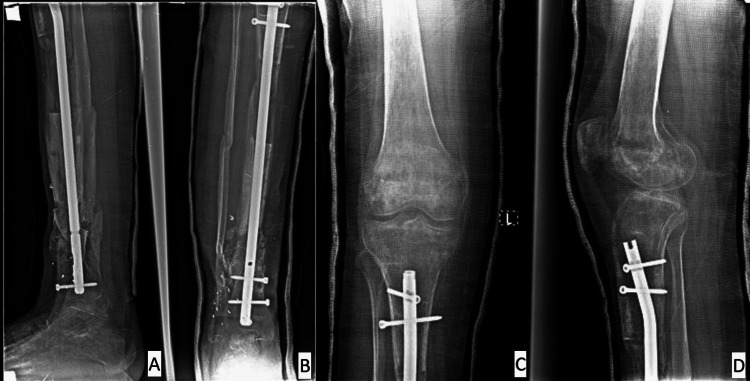
(A-D) Radiographic images illustrating the second-stage procedure, including removal of the Ilizarov external fixator, insertion of a tibial intramedullary nail with proximal and distal locking screws, and placement of an autologous cancellous iliac crest bone graft at the docking site.

The patient was admitted to the orthopedic ward for surgical preparation. Under spinal anesthesia, the left lower limb and the Ilizarov fixator, including all pins, were thoroughly disinfected three times with povidone-iodine. A guidewire was introduced into the tibial canal through a proximal tibial incision (entry point for tibial intramedullary nailing). Flexible reaming of the canal was performed using 8, 9, and 10 mm reamers. A 10-mm-diameter, 26-cm-long titanium tibial intramedullary nail was inserted (Figure 7). The nail was locked proximally with two locking screws and distally with a total of three locking screws (Figure 7) [9].

The Ilizarov external fixator was then completely removed. A small anteromedial incision was made at the distal tibial fracture site, where meticulous surgical debridement was performed to expose healthy bleeding bone ends. An autologous iliac crest bone graft (measuring approximately 1 × 0.5 cm) was harvested from the ipsilateral side and impacted into the fracture gap (Figure 7) [10].

All wounds were closed in layers according to standard surgical protocol. The patient was monitored closely postoperatively in the orthopedic ward.

The patient was discharged home with intravenous dual antibiotic coverage for one week, followed by oral antibiotics for an additional two weeks [11]. Wound dressings were recommended every three days, along with non-weight-bearing instructions for a period of three weeks.

Patient follow-up

The patient was followed clinically and radiologically at three weeks, 1.5 months, three months, six months, and one year postoperatively. Inflammatory markers remained within normal limits, and complete wound healing without signs of infection was achieved by postoperative day 15.

Partial weight-bearing (30%)-walking on the forefoot-was allowed on day 21 following the intramedullary nailing and bone grafting procedure. Full weight-bearing was permitted at six weeks postoperatively [12].

**Figure 8:**
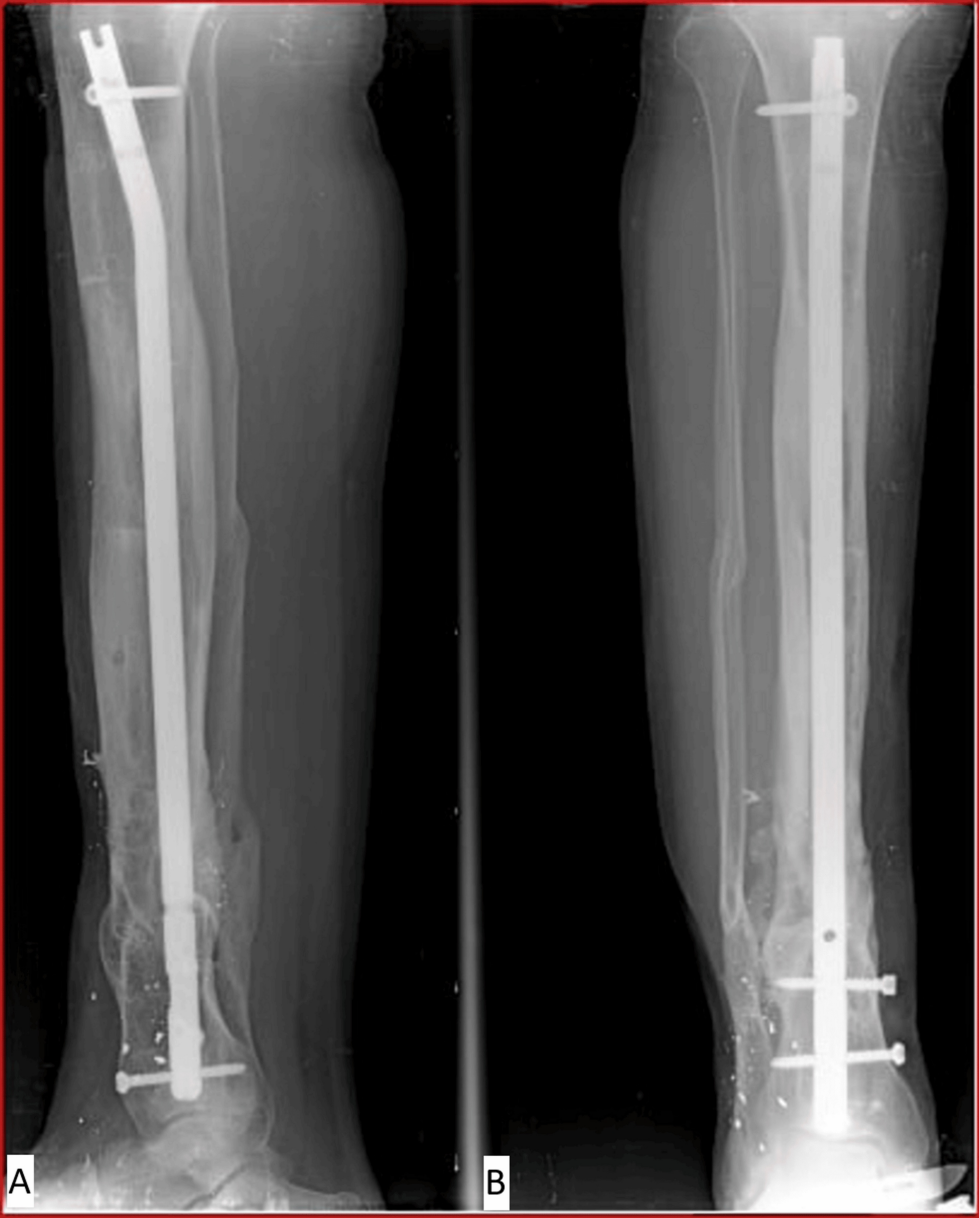
(A, B) Follow-up radiographs obtained six months after the second-stage surgery, demonstrating solid bone union at both the docking site and the distraction regenerate, with satisfactory alignment of the tibia.

Early radiological evidence of satisfactory callus formation was observed approximately three months after bone grafting. Complete union of the bone graft occurred after about six months (Figure 8).

**Figure 9:**
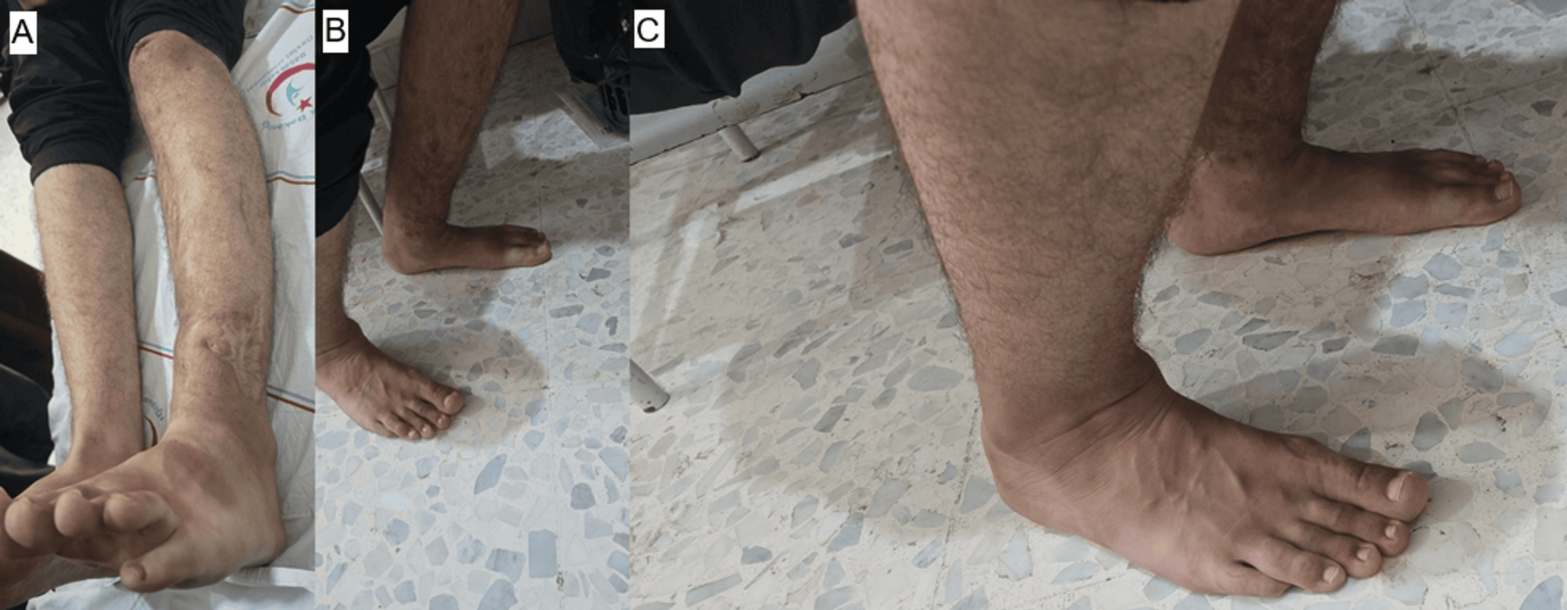
(A-C) Clinical photographs obtained at final follow-up showing restoration of limb length equality and satisfactory functional outcome, including preserved knee and ankle range of motion.

The patient demonstrates an active range of motion of 0° extension and 135° flexion at the knee. At the ankle, active dorsiflexion is 5° and plantarflexion is 15°. Leg lengths are equal (Figure 9).

**Figure 10:**
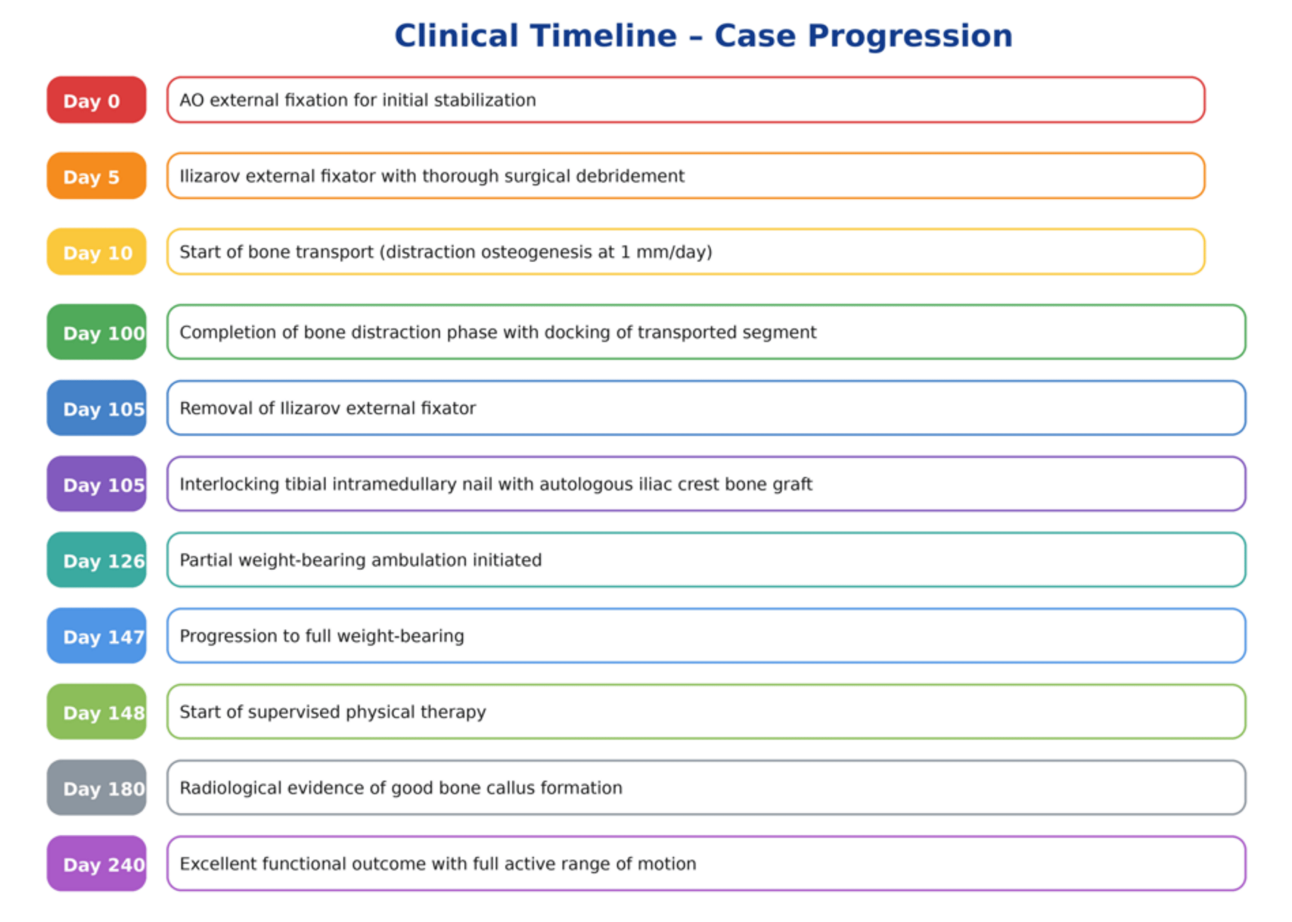
Timeline of clinical management and recovery following a gunshot-induced open tibial fracture.

Finally, the overall treatment course followed a staged limb salvage protocol. Initial damage-control stabilization was achieved using an AO external fixator, followed by application of an Ilizarov circular frame with thorough debridement. Bone transport was initiated after a short latency period, with gradual distraction osteogenesis until docking was completed. Subsequent conversion to an interlocking intramedullary nail with autologous iliac crest bone grafting allowed frame removal and progressive weight-bearing rehabilitation. A detailed timeline of the clinical course is summarized in Figure 10.

## Discussion

War-related injuries are typically high-energy traumas that result in extensive damage to both bone and soft tissues, frequently producing complex open fractures with segmental bone loss. These injuries are associated with a high risk of infection, delayed union, non-union, and chronic osteomyelitis, particularly when initial management is delayed or inadequate [2,13]. Several studies have emphasized that prolonged treatment duration, repeated surgical interventions, and extended use of external fixation significantly contribute to physical disability, psychological distress, and reduced quality of life, especially in young and active patients [7,8].

The management of large post-traumatic tibial bone defects remains challenging, and no single strategy has been universally accepted. Ilizarov distraction osteogenesis has long been regarded as a reliable method for reconstructing segmental bone defects, allowing gradual bone regeneration while addressing infection control and limb length discrepancy [3,7]. However, despite high union rates, multiple reports have highlighted the disadvantages of prolonged circular external fixation, including pin-tract infections, joint stiffness, patient discomfort, and delayed functional recovery [7,8].

To overcome these limitations, hybrid and sequential treatment strategies have gained increasing attention. Wang et al. reported that bone transport combined with sequential intramedullary nailing significantly reduced external fixation time and frame-related complications while maintaining satisfactory union and functional outcomes [14]. Nevertheless, the authors cautioned about the potential risk of infection recurrence, underscoring the importance of careful patient selection, infection surveillance, and thorough patient counseling when adopting this approach. These findings closely align with our strategy of minimizing the interval between external fixation and definitive internal stabilization.

Similarly, recent studies have explored bone transport performed over or followed by intramedullary nailing. Shetu et al. evaluated outcomes of bone transport over an intramedullary nail in long-bone defects and reported high union rates and favorable functional outcomes [15]. However, they also noted common complications such as pin-tract infections and joint stiffness, reinforcing the need to limit the duration of external fixation whenever possible. These observations support our decision to convert early to internal fixation to facilitate rehabilitation and reduce frame-related morbidity.

The role of Ilizarov bone transport in the setting of infection has also been extensively investigated. Alimujiang et al. reported favorable outcomes using Ilizarov bone transport for distal tibial defects following chronic osteomyelitis, demonstrating effective infection control and satisfactory functional recovery even in patients with compromised soft-tissue envelopes [16].

Biological augmentation at the docking site represents another critical factor influencing outcomes. Previous studies have shown that autologous cancellous bone grafting significantly enhances consolidation and reduces the risk of delayed union at the docking site [10]. In our case, bone grafting was performed during the second stage in conjunction with intramedullary nailing, which likely contributed to the timely radiological union observed within six months.

Importantly, a recent meta-analysis by Tan et al. comparing various distraction osteogenesis techniques concluded that while distraction osteogenesis remains highly effective for managing large bone defects, outcomes vary depending on the specific technique employed [17]. The authors emphasized that early conversion to internal fixation, when feasible, may preserve the biological advantages of bone transport while reducing complications associated with prolonged external fixation. This conclusion strongly supports the rationale behind the short-interval two-stage approach used in our case.

Compared with reports describing prolonged circular external fixation alone, the present case highlights the potential advantages of a short-interval two-stage strategy. By limiting the duration of Ilizarov fixation and transitioning early to stable intramedullary fixation with biological augmentation, we achieved reliable bone union, early weight-bearing, and excellent functional recovery without infectious or mechanical complications.

Overall, this case supports and extends current evidence indicating that distraction osteogenesis followed by timely conversion to intramedullary fixation with autologous bone grafting is a safe and effective strategy for managing severe open comminuted tibial fractures with large bone defects. The present report contributes to the growing body of literature advocating for minimizing external fixation time while optimizing both biological healing and functional outcomes.

## Conclusions

This case report describes the successful management of a severe open comminuted tibial diaphyseal fracture with a large segmental bone defect using a short-interval two-stage treatment strategy. In this selected patient, initial distraction osteogenesis enabled reconstruction of the bone defect, followed by timely conversion to intramedullary fixation with autologous bone grafting, which reduced the duration of external fixation and supported functional recovery.

Given the descriptive nature and inherent limitations of a single case report, no definitive conclusions regarding the overall safety, reliability, or superiority of this approach can be drawn. However, this experience suggests that short-interval conversion after bone transport may be a feasible option in carefully selected patients, provided that strict infection control, appropriate timing, and close monitoring are ensured. Further studies involving larger patient cohorts and standardized outcome measures are required to better define the indications, risks, and reproducibility of this strategy, particularly in the context of high-energy and war-related tibial injuries.
